# Patient pathways for rare diseases in Europe: ataxia as an example

**DOI:** 10.1186/s13023-023-02907-y

**Published:** 2023-10-17

**Authors:** Julie Vallortigara, Julie Greenfield, Barry Hunt, Deborah Hoffman, Carola Reinhard, Holm Graessner, Antonio Federico, Vinciane Quoidbach, Steve Morris, Paola Giunti

**Affiliations:** 1 Ataxia Centre, Department of Clinical and Movement Neurosciences, UCL Queen Square Institute of Neurology, Queen Square House, Queen Square, London, WC1N 3BG UK; 2 Ataxia UK, London, UK; 3 Takeda Pharmaceuticals, Cambridge, MA USA; 4 Centre for Rare Diseases and Institute of Medical Genetics and Applied Genomics, University Hospital Tübingen, Tübingen, Germany; 5 Department of Medicine, Surgery and Neurosciences, Medical School, University of Siena, Italy and European Academy of Neurology, Siena, Italy; 6 European Brain Council, Brussels, Belgium; 7 Primary Care Unit, Department of Public Health and Primary Care, University of Cambridge, Cambridge, UK

**Keywords:** Ataxia, Specialist centre, Care pathway, Patient survey, Rare diseases

## Abstract

**Background:**

Progressive ataxias are rare and complex neurological disorders that represent a challenge for the clinicians to diagnose and manage them. This study explored the patient pathways of individuals attending specialist ataxia centres (SAC) compared with non–specialist settings. We investigated specifically how diagnosis was reached, the access to healthcare services, treatments, and care satisfaction. The focus of this study was on early intervention, coordination of treatment to understand the care provision in different countries.

**Methods:**

A patient survey was done in the UK, Germany and Italy to gather information about diagnosis and management of the ataxias in specialist (SAC) and non-specialist settings, utilisation of other primary and secondary health care services, and patients’ satisfaction of received treatment.

**Results:**

Patients gave positive feedback about the role of SAC in understanding their condition, ways to manage their ataxia (p < 0.001; UK) and delivering care adapted to their needs (p < 0.001; UK), in coordinating referrals to other healthcare specialists, and in offering opportunities to take part in research studies. Similar barriers for patients were identified in accessing the SACs among the selected countries, UK, Germany, and Italy.

**Conclusions:**

This study provides crucial information about the ataxia patients care pathways in three European countries. Overall, the results showed a trend in patients’ satisfaction being better in SAC compared to non-SAC. The outcomes can be used now for policy recommendations on how to improve treatment and care for people with these very rare and complex neurological diseases across Europe**.**

**Supplementary Information:**

The online version contains supplementary material available at 10.1186/s13023-023-02907-y.

## Background

The ataxias are a heterogeneous group of chronic progressive neurological disorders, characterized by lack of coordination, difficulty in walking, associated with difficulties in speech, ability to swallow, eye movements, and other symptoms [1]. Gait and balance problems often progress to the point at which patient’s become wheelchair-bound, and the level of disability progresses at the cost of functional independence [2]. Epidemiological studies have estimated an overall ataxia occurrence rate of 26/100,000 in children, and for hereditary cerebellar ataxia an occurrence rate of 2.7–3.3/100,000 [3]. Friedreich’s ataxia, the most common inherited ataxia, has an estimated prevalence of 3.4 cases per 100,000 individuals [4]. Diagnosis is generally a long process because of the rarity and complexity of the different ataxias. Although there are no disease modifying treatments for the majority of progressive ataxias, there are many aspects of the conditions that are treatable, therefore highlighting the importance of guidelines to improve diagnosis and management of the ataxias and associated symptoms [5–7]. The guidelines are aimed at healthcare professionals (HCP) in primary and secondary care (such as general practitioners (GP), general neurologists, clinical geneticists, physiotherapists, speech and language therapists (SLT), occupational therapists (OT)) who provide care for individuals with progressive ataxia and their families [6, 8]. Early intervention in both the diagnosis and management of patients with the ataxia is critical in working towards maintaining functional ability and therefore independence. Specialist ataxia centres (SAC) can provide the necessary clinical expertise and coordination of care and therefore address the specific needs of ataxia patients [9].

This ataxia study aims to examine health gains resulting from specialist healthcare interventions in comparison with non-specialised ones and converge data evidence to policy recommendations on how to improve the care pathway [10]. The management of orphan diseases is increasingly debated within the disease specific networks (European reference networks) as recommended by the EU policy on rare diseases. This is facilitated by the implementation of reference centres or centres of expertise at country level, which are part of the network. The focus of this study is on patient needs, early intervention, and coordination of care and exploring care pathways as a tool to better understand the care provision in different countries. The results have identified treatment gaps and therefore these data can translate into clinical practice guideline recommendations within health care processes, to improve outcomes for patients with rare diseases [11, 12]. The aim was to have a landscape of the rare diseases in different health systems in Europe that will lead to a white paper to improve policy for patients with rare diseases.

## Results

### Demographics

We received 277 responses from participants in the UK, 101 from Germany and 174 from Italy. Respondents were predominantly patients in all three countries. There was an even split in genders (Table 1). Most respondents were 60–80 years of age in the UK whereas the cohorts in Germany and Italy were younger, 30–59 years old (p < 0.001). Participants from the three countries reported being diagnosed with a range of types of ataxias including Friedreich’s ataxia (FRDA), inherited cerebellar ataxia (CA) which include the group of spinocerebellar ataxias, and some people with idiopathic CA. The proportion of participants currently attending a specialist ataxia centre (SAC) was different between the UK and the two other countries. In the UK there were 29% people in YES to SAC group versus 51.4% in NO to SAC group, whereas in Germany and Italy more people were attending SAC, 57.1% in Germany and 59% in Italy (Table 1).

**Table 1:** Survey respondent demographics

A: UK survey		
Respondent type (n = 273)	Patient	234 (85.7%)
	On behalf of the patient	39 (14.3%)
Age distribution (n = 270)	16–29	12 (4.4%)
	30–59	106 (39.3%)
	60–80	140 (51.9%)
	80 +	12 (4.4%)
Gender (n = 270)	Female	142 (52.6%)
	Male	128 (47.4%)
Diagnosis (n = 267)	FRDA	27 (10.1%)
	Inherited CA	78 (29.2%)
	Idiopathic CA	114 (42.7%)
	Other types	38 (14.2%)
	Not known	10 (3.8%)
Attendance to SAC (n = 248)	Never been	128 (51.6%)
	Currently going	72 (29%)
	Used to go	48 (19.4%)

B: Germany survey		
Respondent type (n = 101)	Patient	90 (89.1%)
	On behalf of the patient	11 (10.9%)
Age distribution (n = 101)	16–29	12 (11.9%)
	30–59	60 (59.4%)
	60–80	29 (28.7%)
	80 +	0 (0%)
Gender (n = 101)	Female	48 (47.5%)
	Male	53 (52.5%)
Diagnosis (n = 94)	FRDA	14 (14.9%)
	Inherited CA	52 (55.3%)
	Idiopathic CA	5 (5.4%)
	Other types	10 (10.6%)
	Not known	13 (13.8%)
Attendance to SAC (n = 84)	Never been	23 (23.4%)
	Currently going	48 (57.1%)
	Used to go	13 (15.5%)

C: Italy survey		
Respondent type (n = 173)	Patient	131 (75.7%)
	On behalf of the patient	42 (24.3%)
Age distribution (n = 173)	16–29	22 (12.72%)
	30–59	115 (66.5%)
	60–80	35 (20.2%)
	80 +	1 (0.6%)
Gender (n = 173)	Female	93 (53.8%)
	Male	80 (46.2%)
Diagnosis (n = 159)	FRDA	56 (35.2%)
	Inherited CA	42 (26.4%)
	Idiopathic CA	19 (12%)
	Other types	27 (17%)
	Not known	15 (9.4%)
Attendance to SAC (n = 139)	Never been	27 (19.4%)
	Currently going	82 (59%)
	Used to go	30 (21.6%)

Table A: CA, cerebellar ataxia; FRDA, Friedreich’s ataxia. After data cleaning, there were 277 respondents to the survey. Other types: episodic ataxia, gluten ataxia, hereditary spastic paraplegia, auto-immune ataxias, sensory ataxia

Table B: CA, cerebellar ataxia; FRDA, Friedreich’s ataxia. After data cleaning, there were 101 respondents to the survey. Other types: episodic ataxia, multiple system atrophy cerebellar type C, ataxia with oculomotor apraxia type 2, autoimmune response, leukodystrophy, cerebellar ataxia and mitochondriopathy

Table C: CA, cerebellar ataxia; FRDA, Friedreich’s ataxia. After data cleaning, there were 174 respondents to the survey. Other types: cerebrotendineus xanthomatosis, ataxia with oculomotor apraxia type 1, Gordon Holmes cerebellar ataxia, ataxia due to stiffness, POLR3-related spastic ataxia, Vitamin E deficiency, secondary acquired ataxia, cerebellar ataxia neuropathy and vestibular areflexia syndrome, ataxia post cerebellar cancer, cerebellar ataxia, high probability of post-infectious cause (unknown origin), fragile X associated tremor/ataxia symdrome, spastic ataxia 9, multiple system atrophy, multiple system atrophy cerebellar type C, spastic paraplegia

### Difficulty in reaching a specific diagnosis

Across the three countries, participants who live with an unknown type of ataxia were 46.5% in the UK, 13.8% in Germany and 21.4% in Italy (Table 1). When comparing the proportion of people with specific diagnoses in YES to SAC and NO to SAC groups, there was no difference in proportion of people with specific diagnosis in the UK and in Germany. On the other hand, the Italian cohort showed more specific diagnosis in patients in YES to SAC group compared to NO to SAC group (80% participants in YES to SAC versus 50% in NO to SAC; p < 0.001).

### Referral to SAC

The referral pathway in the three countries was different. In the UK, participants accessed the SAC with a referral from local neurologists, GPs and other HCPs whereas in Germany and Italy the way to access the SAC was through research studies, local neurologists, GPs, and self-referrals (Additional file 1: Table S1).

### Challenges accessing a SAC

The data from people who used to attend a SAC but no longer did (i.e.: the third group identified from the survey) identified several barriers to accessing a SAC. Firstly, respondents stopped going to a SAC as travelling was challenging. The percentage of the respondents who reported this was similar in all the three countries: 25% responses in the UK, 17% in Germany and 27% in Italy (Table 2). Secondly, the lack of a re-referral system to access SAC was another reason mentioned by respondents in all three countries (Table 2). The same reasons for not attending a SAC were identified from the group of patients who never been to a SAC (Additional file 2: Table S2).

**Table 2:** Reasons why people stop going to a SAC to receive care for their ataxia

Reasons	UK N (%)	Germany	Italy
Problems with travelling / transport to SAC	12 (25.5%)	5 (16.7%)	12 (26.6%)
Did not find it useful	6 (12.8%)	1 (3.3%)	8 (17.8%)
Not referred again	7 (14.9%)	1 (3.3%)	-
Equal care locally	2 (4.3%)	4 (13.3%)	8 (17.8%)
Unable to take the time off work to visit the centre	0 (0%)	0 (0%)	0 (0%)
Other [please specify in the text box]	5 (10.6%)	10 (33.4%)	9 (20%)
Unsure	2 (4.3%)	9 (30%)	8 (17.8%)
Do not wish to answer	1 (2.1%)	–	–
Closure of a centre	12 (25.5%)	–	–
Total N respondents	47 (100%)	30 (100%)	45 (100%)

Below are the comments of participants for each country who answer ‘other’: Other reasons in the UK: ataxia is mild, waiting for referral, waiting for an appointment, feel others would benefit more than me i.e. younger people

Other reasons in Germany: cancellation due to Covid, diagnosis not verified so only taking part in research, only required to see them every two years, was asked to see a general neurologist, ongoing care, attended an appointment at SAC as part of a research study

Other reasons in Italy: I am disappointed with the answers from the specialists, Latina is far from Rome, C/O Besta Institute/Milan they told me that at Ferrara hospital they can take care of me, At Besta they couldn't do nothing more, far from the centre - transport problems, I attend a non-ataxia specialist daily association but I am well followed up and they well understand my condition, I am still occasionally followed up, During the first years they seem to help then nothing, I hope they could do more they simply gave me a diagnosis suggesting me to refer to a neurologist I trust, I lost my contacts and not yet found an ataxia specialist who can follow me up in Puglia or near, stopped due to Covid

### Knowledge and understanding of ataxia

When people were asked how well HCPs in primary care services understood how to manage their ataxia, the feedback given was negative for 53% of participants in the UK, 31.5% of participants in Italy and 16% of participants in Germany (Additional file 3: Table S3a). In terms of how well HCPs in primary care knew about the treatments available for ataxia, again some negative feedback was given by 59% of participants in the UK, 30% for Italy and 20% in Germany (Additional file 3: Table S3b).

Feedback was less negative for patients’ experiences of visits to secondary HCPs (including neurologists), compared to their experience of visits in primary care in all three countries. For the understanding of the management of the ataxia, there was negative feedback by 37% of participants in the UK, 5% in Germany, and 18.5% in Italy (Additional file 3: Table S3c). In terms of knowledge about treatments negative feedback was given by 52% of participant in the UK, 3% in Germany, and 20% in Italy (Additional file 3: Table 3d).

**Table 3:** Attendance to a multidisciplinary team clinic including at a non-specialist hospital

Answer choices	UK N (%)	Germany	Italy
Yes	87 (37.8%)	12 (16.45%)	54 (45%)
No	128 (55.7%)	49 (67.1%)	57 (47.5%)
Unsure	15 (6.5%)	12 (16.45%)	9 (7.5%)
Total	230 (100%)	73 (100%)	120 (0%)

Finally, the feedback given by participants about their experience of the visit to a SAC was more positive for both questions: the level of understanding of the management of ataxia and the knowledge about treatments. For the first question, only 9% of participants gave negative feedback in the UK, 0% in Germany and 5% in Italy (Additional file 3: Table 3e-f). In terms of the knowledge on the treatments for ataxia, only 15% participants gave negative feedback in the UK, 0% in Germany and 7% in Italy (Additional file 3: Tables 3e-f).

### Access to a multidisciplinary team (MDT)

Among the three countries, a minority of the respondents received care from an MDT: 38% of participants in the UK; 16% of the respondents in Germany; 45% of the participants in Italy (Table 3). Most of the people who attended an MDT clinic reported positive feedback about the MDT. The proportion of participants who reported that the care received at the MDT was effective was: 88% participants in the UK, 69% participants in Germany, and 66% participants in Italy (Table 4). The survey for Germany and Italy was modified to ask a question about how people were referred to an MDT. Participants from both countries accessed an MDT through various pathways, with a referral made by the specialist at the SAC, the neurologist at standard clinic, or through the GP, physiotherapist, and some self-referral specifically in Italy (Additional file 4: Table S4).

**Table 4:** Feedback of participants on the effectiveness of the multidisciplinary team care they received

Country	UK		Germany			Italy		
Feedback	Positive N (%)	Negative N (%)	Positive N (%)	Neutral N (%)	Negative N (%)	Positive N (%)	Neutral N (%)	Negative N (%)
Number of respondents	74 (88.1%)	10 (11.9%)	11 (68.75%)	5 (31.25%)	0 (0%)	38 (65.5%)	14 (24.1%)	6 (10.4%)
Total respondents	84 (100%)		16 (100%)			58 (100%)		

### Management of the ataxias

We asked participants how well they felt their symptoms were managed overall. Patients in SAC reported a better overall treatment in the UK with 77% people in the YES to SAC group giving positive feedback compared to 45% people in the NO to SAC group; this difference was statistically significant (p < 0.001) (Table 5a). When we asked the participants how well they felt the care they received reflected their needs, in the UK 75% patients in the YES to SAC group reported positive feedback (Table 5b) compared to 52% people in NO to SAC group (p < 0.001); whereas in Germany and in Italy, there was no statistical difference between people in the YES to SAC and NO to SAC groups (Table 5a, b, f). When we asked participants who attended both SAC and non-specialist services in Germany and Italy, if the care at SAC was better than the care in non-SAC, 67% and 40% of the respondents reported that the care was better in the SAC in Germany and Italy respectively (Additional file 5: Table S5). This question was not asked in the UK survey.

**Table 5:** Overall feedback on symptoms management (a) and care received being adapted to people’s needs (b)

Country	UK		Germany		Italy	
(a)						
Feedback on symptom management	Number of respondents in NO to SAC	Number of respondents in YES to SAC	Number of respondents in NO to SAC	Number of respondents in YES to SAC	Number of respondents in NO to SAC	Number of respondents in YES to SAC
Positive N (%)	58 (55%)	48 (77%) ***	15 (88%)	31 (94%)	10 (62.5%)	46 (74%)
Negative N (%)	47 (45%)	14 (23%)***	2 (12%)	2 (6%)	6 (37.5%)	16 (26%)
Total respondents	105 (100%)	62 (100%)	17 (100%)	33 (100%)	16 (100%)	62 (100%)
(b)						
Feedback on care received	Number of respondents in NO to SAC	Number of respondents in YES to SAC	Number of respondents in NO to SAC	Number of respondents in YES to SAC	Number of respondents in NO to SAC	Number of respondents in YES to SAC
Positive N (%)	56 (52%)	48 (75%)***	15 (83%)	30 (85%)	10 (53%)	41 (66%)
Negative N (%)	51 (48%)	16 (25%)***	3 (17%)	5 (15%)	9 (47%)	21 (34%)
Total respondents	107 (100%)	64 (100%)	18 (100%)	35 (100%)	19 (100%)	62 (100%)

^***^p < 0.001. Statistical difference between feedback of SAC and NON SAC groups in the UK for both table a and table b

### Feedback from participants on how to improve their care

The feedback given by participants in the survey from the three different countries on how their diagnosis, treatment and care could be improved is summarised below (Additional file 6: Table S6):

- Earlier specific diagnosis (30.63% for the UK; 21.54% for Germany; 18.33% for Italy);
- Improvement and dissemination of information about the condition (40.54% of participants for UK; 38.46% for Germany; 40% for Italy), and on available treatments (61.71% in the UK; 54.17% in Italy);
- Advice to feel in control of the condition (55.86% for UK; 41.54% for Germany; 53.33% for Italy), practical advice on living with the condition (51.35% for UK; 44.62% for Germany; 46.67% for Italy);
- Better access to therapies (49.55% for UK; 30.77% for Germany; 52.50% for Italy), and better management of their symptoms (39.19% for UK; 33.85% for Germany; 34.17% for Italy);
- More advice on adapting the home (36.94% for UK; 30.77% for Germany; 16.67% for Italy);
- Maintaining the same level of health care when visits to a SAC are not possible anymore (21.17% for UK; 23.08% for Germany; 34.17% for Italy).

## Discussion

Our multinational survey aimed to expand the evidence base on the value of SACs in being able to deliver early coordinated interventions in both diagnosis and management of ataxia patients [13, 14]. This project explores the patient pathways of individuals with different progressive ataxias in a SAC compared with the care in non-specialist settings. To do so, we ran surveys in the UK, Germany, and Italy to collect information about patient’s pathways including diagnosis, treatment, and care of the ataxias. Here we presented a summary of converging data, from the patient survey, collected and analysed for the three selected countries. The aim of this project is to change policy, improving the care pathway for people living with ataxia across Europe.

This study allowed us to collect valuable data on rare neurological disorders in three countries directly from patients about their journeys towards reaching a diagnosis and receiving treatment and care for their ataxia. We have identified some patients’ needs and treatments gaps and now have a better understanding of the value of SACs in the patient care pathways at the European level. Important outcomes of this project are policy recommendations based on converging data collected.

The recruitment of participants was different between countries, namely in Germany and Italy recruitment of participants was not only via patient associations but was also done by clinicians at the SAC, and this was not the case in the UK where recruitment was only via the patient association. This is likely to have had an impact on the cohorts recruited and contributed to the sample population having a higher proportion of people attending SAC in Germany (68%) and Italy (75%) versus UK (36%). Another consequence of this recruitment might be the lower of proportion of patients receiving a specific diagnosis in the UK versus Germany and Italy. In terms of diagnosis, from the data collected we can’t make any conclusions about the role of SACs in reaching a specific diagnosis.

This study has given us a better understanding of the role of SACs in terms of management of the ataxias and care delivered to patients. Participants reported positive feedback on SAC services in terms of the level of understanding of the management of ataxia and the knowledge about treatments in the three countries and this was more positive than responses given to primary or secondary care services generally. Also, participants reported positive feedback for SACs in coordinating referrals to other HCPs in UK and Italy, and better communication with social care professionals in Italy only (Additional file 7: Table S7). The liaison between SAC and social workers is important to help people to get financial support and any other support towards their needs in living with ataxia. This will be particularly helpful when people need care at home, any adaptation their home, when they can no longer attend a SAC (Additional file 6: Table S6).

When participants gave their feedback about overall management of their symptoms and the level of care received being adapted to their needs, there was more positive feedback from people visiting SAC compared to people who have never been to SAC in the UK. This was not the case for Germany and Italy where the feedback was similar whether people went to a SAC or not (Table 5). However, when asked a direct question comparing the overall management of attending an SAC versus a standard of care clinic in Germany there was more positive feedback on the SAC. This was not the case in Italy. This could be due to the different roles SACs play in terms of management of the symptoms versus clinical research activities, with SACs in Italy and Germany focusing more on diagnosis and clinical trials and less on the management (communications from the healthcare professionals who were involved in the survey in these two different countries).

Up to date literature on the ataxia patient care pathway in Europe is scarce. The first step of the care pathway is reaching a specific diagnosis, which can be a challenge considering the rarity of some types of ataxias [15]. An easy access to new techniques for diagnosis including the whole genome sequencing [16] and functional assay is going to provide a higher rate of diagnosis reached and in a timely manner. We published a study on the resource use and burden of Friedreich’s ataxia in the UK and Germany [17], where it is clearly explained how the most common type of ataxia impacts the health and social care of people living with such condition, the caregivers and society. A similar study was done in Spain on cerebellar ataxia showing the costs and impact on patients’ quality of life [18]. There is growing evidence that making a coordinated multidisciplinary care a standard for patients with ataxia would improve the treatment, care, and long-term management of the condition, with the hope to decrease the burden of the disease [19, 20]. Specialist ataxia centres can play such role in delivering knowledge and expertise on ataxia, specific treatments, an adapted care, and coordinating further care needed locally, together with offering opportunities to take part in research studies. One role that Specialist Centres could have is to increase the education of healthcare professionals in primary care to tackle the lack of experience and expertise in the management of the ataxias [6, 8, 21]. One example of such an initiative to educate primary care professionals is a summary of the ataxia guidelines for the management of the ataxias for primary care produced in 2016 by Ataxia UK [22] and published on ‘Guidelines’, the online resource for primary care guidelines in the UK [23]. Another example is the series of educational webinars run by the European Reference Network for Rare Neurological Diseases (ERN-RND) where experts on ataxias have shared their knowledge and expertise on specific aspects of these disorders [24]. In addition, there’s a need to improve appropriate continuous neurorehabilitation and the effort of ERN-RND to promote telerehabilitation protocols for ataxias (Lavorgna L. et al. and Federico A., In preparation). On the other hand, ensuring a liaison between ataxia specialist services and professionals in social care would help to further support patients in their daily quality of life. Despite the efforts of many ataxia specialists in producing ataxia guidelines, such as the Ataxia Guidelines published by members of the UK SACs and others in collaboration with Ataxia UK that were also then endorsed by the ERN-RND, and the educational effort that specialists among the ERN and others are doing, each country also needs to embed the education of rare diseases in its own programme at medical and nursing school level and HCPs continual education training. Then there will be more knowledge and awareness among HCPs about ataxia.

Here we propose some recommendations based on data from our patient survey, on how to improve the care pathway (Table 6).

**Table 6:** Policy recommendations

Survey data	Diagnosis	Access to specialist services	Improve treatment and care		Address gaps in primary care services
	(1) Significant proportion of participants with ataxia of unknown cause (Table 1) (2) reaching a specific diagnosis earlier is important to patients (Additional file 6: Table S6)	(1) Significant proportion of participants have never been to a SAC (Table 1) (2) the access to the SAC is challenging for people with ataxia due to transport involved (Table 2)	(1) A minority of participants accessed an MDT clinic (Table 3) (2) MDT visit was effective for most people who visited an MDT (Table 4)	(1) Participants need to have better information about treatment available for their ataxia, and a better management of their symptoms (Additional file 4: Table S4) (2) SAC can offer opportunity to take part in research (Additional file 4: Table 5)	Participants reported a lack of knowledge and understanding of ataxia from HCP primary care services (Additional file 3: Tables S3a and S3b)
Recommendations	Implement WGS to achieve timely genetic diagnosis	(1) Increase the access to SAC by increasing the awareness of such centres and incorporating referrals to SAC in national care pathways (2) Implement telemedicine in existing centres and increase the number of centres when possible	Increase the availability of MDT clinics. Integrate MDT clinic into the care provision by SAC	Ensure centres have an interest in translational research so treatment will be more evidence based Ensure centres have the capacity to implement management of the symptoms aside other activities	Enhanced educational packages related to the ataxias across primary care services: educational programmes on rare diseases including ataxia for GP and nurses and general HCP training including physiotherapist, SLT, OT

## Conclusions

The ataxias are complex rare neurological disorders with no approved therapies, no disease modifying treatments for most of the patients. The survey has highlighted a pressing need to improve access to specialist ataxia centres as patients feel overall the management is better in that setting compared to primary or secondary settings. From our experience, the collaboration between SACs and ataxia charities has been crucial in their success and a tighter collaboration will facilitate the access to these centres for more patients. Finally, resources should be deployed to support the existing SACs, responding to the increase demand of patients referred to them, implementing telemedicine and to create new SACs where possible.

## Methods

Three countries with existing adult Specialist Ataxia Centres (SAC) were identified for the purpose of this study: UK (two centres), Germany (nine centres), and Italy (eleven centres) (Additional file 9: Appendix 3). A survey was designed to gather patients’ data on experiences of diagnosis and management of their ataxias, whether they were a patient at a SAC or not.

The participation in the survey was open to all patients (or carers of patients, as proxy respondents) with ataxia, who were 16 years old or over. In each country patients' groups or organisations helped with the recruitment of participants. The survey in the UK was mainly disseminated online through the patient group’s channels (mailing list, website, magazine and on social media). Whereas in Germany and Italy it was disseminated via both patients’ groups channels and clinicians working at the SACs.

Ethical approval was obtained for this study in the UK (REC; reference 19/EE/0030). For the two other countries, as the survey was anonymised no ethical approval was needed.

### Survey design

Patient group representatives, a specialist ataxia neurologist, a specialist ataxia nurse, health economists and representatives of pharmaceutical companies were involved in the design of this survey. The final version of the survey questionnaire had 64 non-obligatory questions relating to the following topics: demographics, diagnosis, referrals, patients’ encounters with healthcare professionals, treatments received and patient satisfaction. The context of the study and the medical terms used in the survey were explained at the beginning of the survey (See survey form in Additional file 10: Appendix 1). The survey first rolled out in the UK was subsequently used as a pilot (Additional file 10: Appendix 1), then revised, updated, and translated to be disseminated in Germany and Italy (Additional file 11: Appendix 2).

### Data collection

The survey was rolled out during the following periods: March–May 2019 in the UK, February–October 2020 in Germany, and May–September 2021 in Italy. The data cleaning process involved the removal of all respondents who did not provide informed consent or positive responses to all three screening questions for an anonymised version of the database (questions 3,4,5; see Additional file 10: Appendix 1). Where the respondent gave contradictory responses, the responses to those questions were removed from the analysis. Incomplete surveys were not removed from the analysis, as respondents chose to answer some questions and to skip others. The number of respondents was reported for each individual question.

### Data analysis

Responses to survey questions were stratified by attendance at the SAC. People who had never been to a SAC were grouped in the ‘NO to SAC’ group; people who are currently going to a centre were grouped in ‘YES to SAC’ group. There was a third group of people who used to go to a SAC but no longer go; they were grouped in the ‘USED to SAC’ group. This group was not included in the statistical analysis because they experienced a mixed care pathway. However, the results of the survey for this group have been used to identify barriers to continue to access SACs.

In questions with responses measured on a Likert scale, the responses were grouped into two categories, where affirmative responses were considered to be either `very positive/positive/slightly positive’ or `very effective/effective/slightly effective’ or ‘strongly agree/agree/slightly agree’ and negative responses were considered to be either ‘very negative/negative/slightly negative’ or ‘very ineffective/ineffective/slightly ineffective’ or ‘strongly disagree/disagree/slightly disagree', respectively. For questions 56 to 63, affirmative responses were considered to be ‘best it could be/very well/quite well/adequately’. Results were prepared as tabulated descriptive statistics and presented as numbers (n) and percentage (%) of total respondents per question.

The questions were treated independently, and statistical tests were performed to compare results between the ‘NO to SAC’ and ‘YES to SAC’ groups for each question using the Chi-Square Pearson test.

### Supplementary Information

**Additional file 1. **Referral pathways to attend a SAC

**Additional file 2**. Reasons why people never went to a SAC to receive care for their ataxia

**Additional file 3. a **Participants agreement rate on primary care health professionals (e.g. GP, physiotherapist, occupational therapist) understood how to manage their ataxia. **b** Participants agreement rate on primary care health professionals (e.g. GP, physiotherapist, occupational therapist) understood the treatments available for their ataxia. **c** Participants agreement rate on secondary care health professionals (e.g. neurologist, other consultants at my local hospital) understood how to manage their ataxia. **d** Participants agreement rate on secondary care health professionals (e.g. neurologist, other consultants at my local hospital) understood the treatments available for their ataxia. **e** Participants agreement rate on the specialists at SAC understood how to manage their ataxia. **f** Participants agreement rate on specialists at SAC understood the treatments available for their ataxia.

**Additional file 4**. Referral pathways to attend an MDT.

**Additional file 5**. Feedback on care received in SAC better than care in non-SAC.

**Additional file 6**. Feedback on how to improve the care delivered.

**Additional file 7. **Feedback on SAC services.

**Additional file 8. **Dataset of patients survey for three countries.

**Additional file 9.** List of Specialist Ataxia Centres in the three countries.

**Additional file 10**. Patient survey disseminated in the UK.

**Additional file 11**. Patient survey disseminated in Italy and Germany.

## Data Availability

The data that support the findings of this study are available as an excel file added in the supplementary material at the end of the manuscript (Additional file 8: Data set). In order to conceal anonymity of the participants, we have removed their location.
