# Supplementary material for: Patient pathways for rare diseases in Europe: ataxia as an example
Source: Orphanet J Rare Dis. 2023 Oct 17;18:328. doi: 10.1186/s13023-023-02907-y (PMC10583310; doi:10.1186/s13023-023-02907-y)
Supplement: Supplementary file 1 — Additional file 1. Referral pathways to attend a SAC [file 13023_2023_2907_MOESM1_ESM.docx]

|  | UK N (%) | Germany N (%) | Italy N (%) |
| --- | --- | --- | --- |
| GP | 44 (36.1%) | 17 (26.15%) | 15 (15.5%) |
| Neurologist | 58 (47.5%) | 35 (53.85%) | 14 (14.4%) |
| Other | 19 (15.6%) | 9 (13.85%) | 51 (52.6%) |
| Unsure | 1 (0.8%) | 4 (6.15%) | 17 (17.5%) |
| Total | 122 (100%) | 65 (100%) | 97 (100%) |

Supplementary Table 1: Referral to attend a SAC

Below are the comments of participants for each country who answer ‘other’:

Other ways in the UK: local neurologists, private neurologist, neurologist at SAC through research study, some participants provided information about how they found out and got referred (Ataxia UK information), Ear Nose Throat consultant.

Other ways in Germany: self-arrangement due to EFACTS (European Friedreich's Ataxia Consortium for Translational Studies), self-referral with ataxia in the family, personal connections, neurologist with own practice, mother with ataxia and took part in the study for non-examined relatives, GP referred in according to recommendation by Prof Schoels, social-paediatrist centre, was interviewed by SCA3 runs in the family, no referral as arranged appointment myself, heard of the clinic from relatives and arranged an appointment myself.

Other ways in Italy: on my own, a cousin perhaps, voluntarily for RG283 testing, psychiatrist colleague, GP first and then Hospital neurologist, Orthopaedist, a friend, telethon operator, Physiatrist, Neurologist, Neurologist /private clinic, on my own, I requested for an appointment at Besta Institute in Milan, speaking with physiotherapist, Association, We saw AISA tv presentation linked to the event " la Vela per la Vita" speaking about ataxia and Besta Institute in Milan.
