# Supplementary material for: Patient pathways for rare diseases in Europe: ataxia as an example
Source: Orphanet J Rare Dis. 2023 Oct 17;18:328. doi: 10.1186/s13023-023-02907-y (PMC10583310; doi:10.1186/s13023-023-02907-y)
Supplement: Supplementary file 2 — Additional file 2. Reasons why people never went to a SAC to receive care for their ataxia [file 13023_2023_2907_MOESM2_ESM.docx]

| Reasons | UK N (%) | Germany N (%) | Italy N (%) |
| --- | --- | --- | --- |
| Current level of care is sufficient | 18 (10.3%) | 10 (18.2%) | 6 (8%) |
| I asked to be referred to a SAC but was refused by my doctor | 6 (3.4%) | 0 (0%) | 0 (0%) |
| SACs are too far away for me to travel to | 29 (16.6%) | 3 (5.4%) | 11 (14.7%) |
| Did not wish to be referred | 2 (1.1%) | 0 (0%) | 6 (8%) |
| A referral to SAC was not offered to me | - | 10 (18.2%) | 20 (26.7%) |
| Other [please specify in the text box] | 24 (13.7%) | 8 (14.5%) | 4 (5.3%) |
| Not applicable | 78 (44.6%) | 20 (36.4%) | 22 (29.3%) |
| Unsure | 17 (9.7%) | 4 (7.3%) | 6 (8%) |
| Do not wish to answer | 1 (0.6%) | - | - |
| Total N respondents | 175 (100%) | 55 (100%) | 75 (100%) |

Supplementary Table 2: Reasons why people never went to a SAC to receive care for their ataxia

Below are the comments of participants for each country who answer ‘other’:

Other reasons in the UK: issues with travelling, not aware of centres, never been mentioned as an option, I have never asked to be referred, still waiting for referral, never been referred, managing the care locally, not worth it (time/distance/cost)

Other reasons in Germany: I was seen at a specialist centre to be genetically tested, diagnosis in progress, visited another ataxia clinic (Göttingen), I am visiting the one in Frankfurt. This one is not on the list, I was referred to a specialist ataxia centre, They're probably overstressed, It was also never offered, The disease cannot be cured. I don't need to know how far the disease has progressed, Immediate diagnosis at UKSH. Offered to participate in a clinical study but they didn't respond yet. I asked several times but they never answered. Apparently, SCA4 is not rare enough and though not 'profitable' enough to research on it.

Other reasons in Italy: The neurologist at Besta Institute in Milan collaborates with the specialist ataxia centre but he didn't consider it useful to send the patient to his colleagues, Covid arrived immediately after the diagnosis, too far.
