# Supplementary material for: Patient pathways for rare diseases in Europe: ataxia as an example
Source: Orphanet J Rare Dis. 2023 Oct 17;18:328. doi: 10.1186/s13023-023-02907-y (PMC10583310; doi:10.1186/s13023-023-02907-y)
Supplement: Supplementary file 3 — Additional file 3. a Participants agreement rate on primary care health professionals (e.g. GP, physiotherapist, occupational therapist) understood how to manage their ataxia. b Participants agreement rate on primary care health professionals (e.g. GP, physiotherapist, occupational therapist) understood the treatments available for their ataxia. c Participants agreement rate on secondary care health professionals (e.g. neurologist, other consultants at my local hospital) understood how to manage their ataxia. d Participants agreement rate on secondary care health professionals (e.g. neurologist, other consultants at my local hospital) understood the treatments available for their ataxia. e Participants agreement rate on the specialists at SAC understood how to manage their ataxia. f Participants agreement rate on specialists at SAC understood the treatments available for their ataxia. [file 13023_2023_2907_MOESM3_ESM.docx]

Supplementary Table 3a: Participants agreement rate on primary care health professionals (e.g. GP, physiotherapist, occupational therapist) understood how to manage their ataxia

| Country | UK | | Germany | | | Italy | | |
| --- | --- | --- | --- | --- | --- | --- | --- | --- |
| Feedback | Positive N (%) | Negative N (%) | Positive N (%) | Neutral  N (%) | Negative N (%) | Positive N (%) | Neutral  N (%) | Negative N (%) |
| YES to SAC | 32 (53.3%) | 28 (46.7%) | 16 (41%) | 16 (41%) | 7(18%) | 31 (48.4%) | 16 (25%) | 17 (26.6%) |
| NO to SAC | 44  (45.4%) | 53 (54.6%) | 6 (40%) | 7 (47%) | 2 (13%) | 10 (41.7%) | 4 (16.6%) | 10 (41.7%) |
| USED to SAC | 19 (43.2%) | 25 (56.8%) | 5 (62.5%) | 2 (25%) | 1 (12.5%) | 7 (30.4%) | 8 (34.8%) | 8 (34.8%) |
| Total | 95 (47.3%) | 106 (52.7%) | 27 (43.5%) | 25 (40.3) | 10 (16.2%) | 48 (43.2%) | 28 (25.2%) | 35 (31.5%) |
| Total respondents | 201 | | 62 | | | 111 | | |

Supplementary Table 3b: Participants agreement rate on primary care health professionals (e.g. GP, physiotherapist, occupational therapist) understood the treatments available for their ataxia

| Country | UK | | Germany | | | Italy | | |
| --- | --- | --- | --- | --- | --- | --- | --- | --- |
| Feedback | Positive N (%) | Negative N (%) | Positive N (%) | Neutral  N (%) | Negative N (%) | Positive N (%) | Neutral  N (%) | Negative N (%) |
| YES to SAC | 26 (44.1%) | 33 (55.9%) | 16 (43.3%) | 13 (35.1%) | 8 (21.6%) | 25 (40.3%) | 17 (27.4%) | 20 (32.3% |
| NO to SAC | 38  (40.0%) | 57 (60.0%) | 6 (31.6%) | 8 (42.1%) | 5 (26.3%) | 16 (59.3%) | 5 (18.5%) | 6 (22.2%) |
| USED to SAC | 17 (39.5%) | 26 (60.5%) | 6 (66.7%) | 3 (33.3%) | 0 (0%) | 9 (37.5%) | 7 (29.2%) | 8 (33.3%) |
| Total | 81 (41%) | 116 (59%) | 28 (43%) | 24 (37%) | 13  (20%) | 50 (44.2%) | 29 (25.7%) | 34 (30.1%) |
| Total respondents | 197 | | 65 | | | 113 | | |

Supplementary Table 3c: Participants agreement rate on secondary care health professionals (e.g. neurologist, other consultants at my local hospital) understood how to manage their ataxia

| Country | UK | | Germany | | | Italy | | |
| --- | --- | --- | --- | --- | --- | --- | --- | --- |
| Feedback | Positive N (%) | Negative N (%) | Positive N (%) | Neutral  N (%) | Negative N (%) | Positive N (%) | Neutral  N (%) | Negative N (%) |
| YES to SAC | 42 (68.9%) | 19 (31.1%) | 24 (68.55%) | 8 (22.9%) | 3 (8.55%) | 33 (55.9%) | 18 (30.5%) | 8 (13.6%) |
| NO to SAC | 56  (58.3%) | 40 (41.7%) | 14 (82.4%) | 3 (14.6%) | 0 (0%) | 7 (31.8%) | 7 (31.8%) | 8 (36.4%) |
| USED to SAC | 29 (65.9%) | 15 (34.1%) | 8 (100%) | 0 (0%) | 0 (0%) | 9 (40.9%) | 10 (45.5%) | 3 (13.6%) |
| Total | 127 (63.2%) | 74 (36.8%) | 46 (76.7%) | 11 (18.3%) | 3 (5%) | 49 (47.5%) | 35 (34%) | 19 (18.5%) |
| Total respondents | 201 | | 60 | | | 103 | | |

Supplementary Table 3d: Participants agreement rate on secondary care health professionals (e.g. neurologist, other consultants at my local hospital) understood the treatments available for their ataxia

| Country | UK | | Germany | | | Italy | | |
| --- | --- | --- | --- | --- | --- | --- | --- | --- |
| Feedback | Positive N (%) | Negative N (%) | Positive N (%) | Neutral  N (%) | Negative N (%) | Positive N (%) | Neutral  N (%) | Negative N (%) |
| YES to SAC | 26 (50.0%) | 26 (50.0%) | 23 (67.6%) | 9 (26.5) | 2 (5.9%) | 33 (55.9%) | 17 (28.8%) | 9 (15.3%) |
| NO to SAC | 43  (47.8%) | 47  (52.2%) | 14 (87.5%) | 2 (12.5%) | 0 (0%) | 8 (36.4%) | 6 (27.3%) | 8 (36.4%) |
| USED to SAC | 18 (47.4%) | 20 (52.6%) | 8 (100%) | 0 (0%) | 0 (0%) | 7 (36.8%) | 9 (47.4%) | 3 (15.8%) |
| Total | 87 (48.3%) | 93 (51.7%) | 45 (77.6) | 11 (19%) | 2 (3.4%) | 48 (48%) | 32 (32%) | 20 (20%) |
| Total respondents | 180 | | 58 | | | 100 | | |

Supplementary Table 3e: Participants agreement rate on the specialists at SAC understood how to manage their ataxia

| Country | UK | | Germany | | | Italy | | |
| --- | --- | --- | --- | --- | --- | --- | --- | --- |
| Feedback | Positive N (%) | Negative N (%) | Positive N (%) | Neutral  N (%) | Negative N (%) | Positive N (%) | Neutral  N (%) | Negative N (%) |
| YES to SAC | 61 (96.8%) | 2  (3.2%) | 38 (95%) | 2 (5%) | 0 (0%) | 55 (78.6%) | 12 (17.1%) | 3 (4.3%) |
| USED to SAC | 31 (81.6%) | 7  (18.4%) | 7 (100%) | 0 (0%) | 0 (0%) | 16 (69.6%) | 5 (21.7%) | 2 (8.7%) |
| Total | 92 (91%) | 9 (9%) | 45 (95.7%) | 2 (4.3%) | 0 (0%) | 71 (76.3%) | 17 (18.3%) | 5 (5.4%) |
| Total respondents | 101 | | 47 | | | 93 | | |

Supplementary Table 3f: Participants agreement rate on specialists at SAC understood the treatments available for their ataxia

| Country | UK | | Germany | | | Italy | | |
| --- | --- | --- | --- | --- | --- | --- | --- | --- |
| Feedback | Positive N (%) | Negative N (%) | Positive N (%) | Neutral  N (%) | Negative N (%) | Positive N (%) | Neutral  N (%) | Negative N (%) |
| YES to SAC | 54  (93.1%) | 4  (6.9%) | 38 (95%) | 2 (5%) | 0 (0%) | 54 (79.4%) | 10 (14.7%) | 4 (5.9%) |
| USED to SAC | 25 (71.4%) | 10 (28.6%) | 7 (100%) | 0 (0%) | 0 (0%) | 15 (65.2%) | 6 (26.1%) | 2 (8.7%) |
| Total | 79 (84.9%) | 14 (15.1%) | 45 (95.7%) | 2 (4.3%) | 0 (0%) | 69 (75.8%) | 16 (17.6%) | 6 (6.6%) |
| Total respondents | 93 | | 47 | | | 91 | | |
