# Supplementary material for: Patient pathways for rare diseases in Europe: ataxia as an example
Source: Orphanet J Rare Dis. 2023 Oct 17;18:328. doi: 10.1186/s13023-023-02907-y (PMC10583310; doi:10.1186/s13023-023-02907-y)
Supplement: Supplementary file 4 — Additional file 4. Referral pathways to attend an MDT. [file 13023_2023_2907_MOESM4_ESM.docx]

Supplementary Table 4: Referral to attend an MDT

|  | Germany N (%) | Italy N (%) |
| --- | --- | --- |
| Neurologist at SAC | 1 (9.1%) | 24 (39.34%) |
| Neurologist (non-specialist clinic) | 5 (45.45%) | 24 (39.34%) |
| Other | 5 (45.45%) | 13 (21.31%) |
| Total | 11 (100%) | 61 (100%) |

Below are the comments of participants for each country who answer ‘other’:

Other ways in Germany: GP referral to physiotherapy, My GP referred me to an inpatient rehabilitation, GP, Feldenkrais physiotherapy, GP, Multidisciplinary Team at rehab clinic, GP.

Other ways in Italy: Physiatrist, AISA, Ataxia Centre neurologist, physiotherapist, partner, myself, my own research of the Multidisciplinary team, my wife asked for physiatrist outpatient visit and activated physiotherapy and speech therapy, GP, geneticist, AISA in Lazio, friends.
