# Supplementary material for: Patient pathways for rare diseases in Europe: ataxia as an example
Source: Orphanet J Rare Dis. 2023 Oct 17;18:328. doi: 10.1186/s13023-023-02907-y (PMC10583310; doi:10.1186/s13023-023-02907-y)
Supplement: Supplementary file 5 — Additional file 5. Feedback on care received in SAC better than care in non-SAC. [file 13023_2023_2907_MOESM5_ESM.docx]

Supplementary Table 5: Feedback on care received in SAC better than care in non-SAC

|  | Germany N (%) | Italy N (%) |
| --- | --- | --- |
| Yes | 43 (67.2%) | 46 (40.4%) |
| No | 4 (6.2%) | 39 (34.2%) |
| Non applicable | 6 (9.4%) | 20 (17.5%) |
| Unsure | 11 (17.2%) | 9 (7.9%) |
| Total | 64 (100%) | 114 (100%) |
