# Supplementary material for: Patient pathways for rare diseases in Europe: ataxia as an example
Source: Orphanet J Rare Dis. 2023 Oct 17;18:328. doi: 10.1186/s13023-023-02907-y (PMC10583310; doi:10.1186/s13023-023-02907-y)
Supplement: Supplementary file 6 — Additional file 6. Feedback on how to improve the care delivered. [file 13023_2023_2907_MOESM6_ESM.docx]

Supplementary Table 6: Feedback on how to improve the care delivered

**UK**

| **Answer choices** | **Responses N** | **Responses (%)** |
| --- | --- | --- |
| More information about my condition | 90 | 40.54 |
| More information about available treatments | 131 | 61.71 |
| More help so I can feel in control of my disease (to cope better) | 121 | 55.86 |
| Knowing my specific diagnosis earlier | 68 | 30.63 |
| Better management of my symptoms | 87 | 39.19 |
| Better practical advice on living with my condition | 114 | 51.35 |
| Better access to therapies (physiotherapy, speech therapy, occupational therapy) | 110 | 49.55 |
| More information on help adapting my home | 82 | 36.94 |
| Help in communicating with my employer | 14 | 6.31 |
| More information about the genetics of my condition/ whether my children or grandchildren are at risk of inheriting ataxia | 59 | 26.58 |
| Continuing the same level of care in my home if I am not longer able to visit an ataxia specialist centre | 47 | 21.17 |
| I am satisfied with my care and do not need improvement | 19 | 8.56 |
| Unsure | 7 | 3.15 |
| I do not know | 1 | 0.45 |
| Total number of respondents | 242 |  |

**Germany**

| **Answer choices** | **Responses N** | **Responses (%)** |
| --- | --- | --- |
| More information about my condition | 25 | 38.46 |
| More help so I can feel in control of my disease (to cope better) | 27 | 41.54 |
| Knowing my specific diagnosis earlier | 14 | 21.54 |
| Better management of my symptoms | 22 | 33.85 |
| Better practical advice on living with my condition | 29 | 44.62 |
| Better access to therapies (physiotherapy, speech therapy, occupational therapy) | 20 | 30.77 |
| More information on help adapting my home | 20 | 30.77 |
| Help in communicating with my employer | 7 | 10.77 |
| More information about the genetics of my condition/ whether my children or grandchildren are at risk of inheriting ataxia | 10 | 15.38 |
| Continuing the same level of care in my home if I am not longer able to visit an ataxia specialist centre | 15 | 23.08 |
| I am satisfied with my care and do not need improvement | 9 | 13.85 |
| Other please specify | 7 | 10.77 |
| I do not know | 1 | 1.54 |

Comments: Similar feedback compared to what was said by patients in the UK: improve information about the disease, treatment, feeling more in control, advice on how to cope better, better access to therapies, adaptation to the home is also important here, local care.

**Italy**

| **Answer choices** | **Responses N** | **Responses (%)** |
| --- | --- | --- |
| More information about my condition | 48 | 40 |
| More information on available treatments | 65 | 54.17 |
| Better management of my symptoms | 64 | 53.33 |
| More help so I can feel in control of my disease (to cope better) | 22 | 18.33 |
| Knowing my specific diagnosis earlier | 41 | 34.17 |
| Better practical advice on living with my condition | 56 | 46.67 |
| Better access to therapies (physiotherapy, speech therapy, occupational therapy) | 63 | 52.50 |
| More information on help adapting my home | 20 | 16.67 |
| Help in communicating with my employer | 7 | 5.83 |
| More information about the genetics of my condition/ whether my children or grandchildren are at risk of inheriting ataxia | 20 | 16.67 |
| Continuing the same level of care in my home if I am not longer able to visit an ataxia specialist centre | 41 | 34.17 |
| I am satisfied with my care and do not need improvement | 7 | 5.83 |
| Other please specify | 4 | 3.33 |
| I am not sure | 0 | 0.00 |

Comments: I need a specific diagnosis,

I have been quite well followed up and against ataxia there is little to do, better practical advice on living with my condition, overall, I wish to get-in real time - more information about ataxia possible treatments, make specific trainings to general practitioners, to Md and Healthcare workers in Accident & Emergency mandatory.
