# Supplementary material for: Patient pathways for rare diseases in Europe: ataxia as an example
Source: Orphanet J Rare Dis. 2023 Oct 17;18:328. doi: 10.1186/s13023-023-02907-y (PMC10583310; doi:10.1186/s13023-023-02907-y)
Supplement: Supplementary file 7 — Additional file 7. Feedback on SAC services. [file 13023_2023_2907_MOESM7_ESM.docx]

Table 7: feedback on SAC services

| **UK**  Group N (%) | YES to SAC | | | Used to SAC | | | Total | | |
| --- | --- | --- | --- | --- | --- | --- | --- | --- | --- |
| Feedback | Positive | Negative | | positive | Negative | | Positive | Negative | |
| Coordination of referrals to specialists | 58 (86.6) | | 9 (13.4) | 26 (61.9) | | 16 (38.1) | 84 | | 25 |
| Total | 67 (100) | | | 42 (100) | | | 109 | | |
| Offers to participate in research | 46 (85.2) | | 8 (14.8) | 24 (60) | | 16 (40) | 70 | | 24 |
| Total | 54 (100) | | | 40 (100) | | | 94 | | |
| Help with securing parking badges | 20 (62.5) | | 12 (37.5) | 19 (65.5) | | 10 (34.5) | 39 | | 22 |
| Total | 32 (100) | | | 29 (100) | | | 61 | | |
| Help with benefits | 12 (40) | | 18 (60) | 13 (40.4) | | 19 (59.6) | 25 | | 37 |
| Total | 30 (100) | | | 32 (100) | | | 62 | | |
| Liaising with social workers | 6 (25) | | 18 (75) | 8 (28.6) | | 20 (71.4) | 14 | | 38 |
| Total | 24 (100) | | | 28 (100) | | | 52 | | |

In the UK: Positive feedback from patients attending the SAC in terms of coordinating referrals, offers to research and help with parking badges

Negative feedback about liaising with social workers and help to get benefits

| **Germany**  Group N (%) | YES to SAC | | | | | | Used to SAC | | | | Total | | | | |
| --- | --- | --- | --- | --- | --- | --- | --- | --- | --- | --- | --- | --- | --- | --- | --- |
| Feedback | Positive | | Neutral | Negative | | | positive | Neutral | Negative | | Positive | | Neutral | Negative | |
| Coordination of referrals to specialists | 19 (50) | | 17(44.7) | 2 (5.3) | | | 1(12.5) | 6 (75) | 1 (12.5) | | 20 (43.5) | 23 (50) | | 3 (6.5) | |
| Total | 38 (100) | | | | | | 8 (100) | | | | 46 (100) | | | | |
| Offers to participate in research | 34 (85) | 5 (12.5) | | | | 1 (2.5) | 4 (50) | 3 (37.5) | | 1 (2.5) | 38 (79.15) | 8 (16.65) | | | 2 (4.15) |
| Total | 40 (100) | | | | | | 8 (100) | | | | 48 (100) | | | | |
| Liaising with social workers | 16 (44.4) | 20 (55.6) | | | 0 (0) | | 2 (28.6) | 4 (57.1) | | 1 (14.3) | 18 (41.9) | 24 (55.8) | | | 1 (2.3) |
| Total | 36 (100) | | | | | | 7 (100) | | | | 43 (100) | | | | |

In Germany:

SAC do better in offering opportunities to take part in research

BUT not better in coordinating referrals and communications with social care professionals

| **Italy**  Group N (%) | YES to SAC | | | | | | | | Used to SAC | | | | | | Total | | | | | | |
| --- | --- | --- | --- | --- | --- | --- | --- | --- | --- | --- | --- | --- | --- | --- | --- | --- | --- | --- | --- | --- | --- |
| Feedback | Positive | | Neutral | | Negative | | | | positive | Neutral | | Negative | | | Positive | | Neutral | | Negative | | |
| Coordination of referrals to specialists | 47 (66.2) | | 21 (29.6) | | 3 (4.2) | | | | 9 (39.1) | 13 (56.5) | | 1 (4.4) | | | 56 (59.6) | 34 (36.2) | | | 4 (4.3) | | |
| Total | 71 (100) | | | | | | | | 23 (100) | | | | | | 94 (100) | | | | | | |
| Offers to participate in research | 60 (85.7) | 8 (11.4) | | | | | 2 (2.9) | | 9 (45) | 11 (55) | | | 0 (0) | | 69 (76.7) | 19 (21.1) | | | | 2 (2.2) | |
| Total | 70 (100) | | | | | | | | 20 (100) | | | | | | 90 (100) | | | | | | |
| Help with benefits | 26 (38.2) | | | 35 (51.5) | | | | 7 (10.3) | 8 (40) | | 11 (55) | | | 1 (5) | 34 (38.6) | | | 46 (52.3) | | | 8 (9.1) |
| Total | 68 (100) | | | | | | | | 20 (100) | | | | | | 88 (100) | | | | | | |
| Liaising with social workers | 38 (53.5) | 30 (42.3) | | | | 3 (4.2) | | | 12 (60) | 7 (35) | | | 1 (5) | | 50 (54.9) | 37 (40.7) | | | | 4 (4.4) | |
| Total | 71 (100) | | | | | | | | 20 (100) | | | | | | 91 (100) | | | | | | |

In Italy: SAC do better in coordinating referrals and offering opportunities to take part in research, communications with social care professionals.

BUT not better in helping to receive benefits
