# Supplementary material for: Patient pathways for rare diseases in Europe: ataxia as an example
Source: Orphanet J Rare Dis. 2023 Oct 17;18:328. doi: 10.1186/s13023-023-02907-y (PMC10583310; doi:10.1186/s13023-023-02907-y)
Supplement: Supplementary file 9 — Additional file 9. List of Specialist Ataxia Centres in the three countries. [file 13023_2023_2907_MOESM9_ESM.docx]

List of centres currently active:

**UK: two centres**

- Royal Hallamshire Hospital in Sheffield (led by Professor Marios Hadjivassiliou),
- National Hospital for Neurology and Neurosurgery in London (led by Professor Giunti)

**Germany: nine centres**

- Friedrich-Baur-Institut am Klinikum der Universität München (Prof. Dr. Thomas Klopstock, Dr. Ivan Karin)
- Universitätsklinikum Tübingen (Prof. Dr. Ludger Schöls, Prof. Dr. Matthis Synofzik)
- Universitätsklinikum Bonn (Prof. Dr. Thomas Klockgether)
- Universitätsklinikum Schleswig-Holstein (Lübeck) (Prof. Dr. med. A. Münchau, PD Dr. med. Y. Hellenbroich, Prof. Dr. med. N. Brüggemann)
- Universitätsklinikum Essen (Prof. Dr. med. D. Timmann-Braun)
- Universitätsklinikum Aachen (Univ.-Prof. Dr. med. Kathrin Reetz, Dr. med. Florian Holtbernd)
- Charité, Universitätsklinikum Berlin (Prof. Dr. med. Stephan Brandt, Dr. Sarah Doss, Maria Rönnefarth)
- Universitätsklinikum Düsseldorf (PD Dr. med.
- Martina Minnerop)
- Universtitätsklinik für Neurologie Magdeburg (Prof. Dr. med. Stefan Vielhaber)

**Italy: eleven centres**

- FIRENZE, Clinica Neurologica, Policlinico, Università degli Studi; DOTT.SSA PIACENTINI SILVIA sostituita da Dott.ssa Camilla Ferrari
- MILANO, IRCCS, Istituito Neurologico “C. Besta”; DOTT.SSA MARIOTTI CATERINA
- E DOTT. TARONI FRANCO
- MESSINA, Policlinico, Università degli Studi; DOTT.SSA MUSUMECI OLIMPIA
- NAPOLI, AOU Federico II, Clinica Neurologica, Università degli Studi; PROF FILLA ALESSANDRO e PROF SACCA’ FRANCESCO
- ROMA, Università degli Studi, La Sapienza, Polo Latina; DOTT. CASALI CARLO
- ROMA, IRCCS, Bambin Gesù; DOTT. BERTINI ENRICO
- SIENA, Policlinico, Le Scotte, Università degli Studi; Prof. ANTONIO FEDERICO
- TORINO, SC Neurologia- AOU Città della Salute e della Scienza di Torino; DOTT. BRUSCO ALFREDO e DOTT.SSA ORSI LAURA
- PISA IRCCS Stella Maris PROF. SANTORELLI FILIPPO
- GENOVA IRCCS Ospedale Policlinico San Martino DOTT. FANCELLU ROBERTO
- BOLOGNA Centro il BeNe Dott. SALVI FABRIZIO
