# Supplementary material for: Patient pathways for rare diseases in Europe: ataxia as an example
Source: Orphanet J Rare Dis. 2023 Oct 17;18:328. doi: 10.1186/s13023-023-02907-y (PMC10583310; doi:10.1186/s13023-023-02907-y)
Supplement: Supplementary file 10 — Additional file 10. Patient survey disseminated in the UK. [file 13023_2023_2907_MOESM10_ESM.docx]

Ataxia UK Patient Care Pathways Survey

Thank you for taking the time to complete and submit this survey. Please take time to read the participant information sheet provided alongside this survey carefully, and feel free to discuss it with others, such as your family and friends.

The purpose of this study is to gather the experiences of care of people with ataxia in the UK, in order to understand ataxia patients’ care pathways and history of accessing specialist ataxia centres in the UK.

You may wish to discuss your answers to the survey with your parents if you were diagnosed when you were very young. Parents/carers/guardians filling in the survey should respond as per the patient’s opinion, not your own.

### Personal details and confidentiality

All the information you provide during the course of this study will be kept confidential. Only the appointed research team will have direct access to your name and email address, if you provide them, and your survey responses. The survey can be completed anonymously if you wish, ensuring that nobody taking part or working on the study will be able to identify you from your survey responses, unless you indicate that you are willing to be contacted for future research and/or would like to receive a copy of the study results via email at the end of the study.

Your survey responses will be combined with responses from other patients in the study, to allow analyses to be carried out by the appointed research team. This way, it will not be possible to identify any single patient from the pooled survey response data. All data will be stored in a form protected by passwords so only people working on the study will be able to access it, and will be deleted 6 months after study analyses are complete.

### Organisation and funding of the study

This study has been sponsored by the European Brain Council (EBC), is funded by Takeda Pharmaceutical Company Ltd and Reata Pharmaceuticals Inc and is being carried out by the charity Ataxia UK, University College London and Costello Medical, a UK-based healthcare consultancy, who are providing scientific support with the study.

### Contact information

Should you wish to ask any further questions about the study before, during or after it has been completed, or if you have any concerns, please contact Julie Greenfield:

Email address: jgreenfield@ataxia.org.uk

Telephone: 020 7582 1444

Address: Ataxia UK. 12 Broadbent Close, London, N6 5JW.

### Completing the study

Please answer all questions, unless indicated otherwise, by ticking the relevant box or by writing your answer.

Please return your completed survey in the envelope provided to Ataxia UK, 12 Broadbent Close, London, N6 5JW, United Kingdom.

Definitions of specific terms used in the survey are provided below:

| **Term** | **Definition** |
| --- | --- |
| General ataxia diagnosis | The first time that a healthcare provider told you that you had ataxia. |
| Specific ataxia diagnosis | When a healthcare provider told you that you that you had a particular type of ataxia, e.g. spinocerebellar ataxia type 1. Some people completing this survey will not have a specific ataxia diagnosis.  For the purposes of this survey, idiopathic ataxia syndrome and cerebellar ataxia (unknown cause) are not considered specific diagnoses. |
| Specialist ataxia centre (SAC) | A centre that specialises in care of ataxia patients only; Royal Hallamshire Hospital in Sheffield (led by Professor Marios Hadjivassiliou), National Hospital for Neurology and Neurosurgery in London (led by Professor Giunti), the Royal Victoria Infirmary in Newcastle (was led by neurologists Professor Patrick Chinnery and Professor Rita Horvath but this centre closed in September 2018).  There are other doctors around the country with an interest in ataxia, but this survey is specifically considering specialist ataxia centres, and these doctors should not be considered as specialist ataxia centres. |
| Primary care | Healthcare delivered outside hospitals. It includes a range of services, including community clinics, health centres and walk-in centres, and is delivered by primary care professionals (please see below). |
| Primary care professional | Someone who provides medical care outside of a hospital, e.g. a general practitioner (GP), nurse practitioner, pharmacist, health visitor, midwife, dentist, or optician. |
| Secondary care | Healthcare provided in hospitals. This includes accident and emergency departments, outpatient departments, antenatal services, genitourinary medicine and sexual health clinics. |
| Inpatient stay | An overnight admission to a hospital. |
| Outpatient visit | A visit to a hospital to undergo tests or receive same-day surgery. |
| Tertiary care | Care for people needing complex treatments. People may be referred for tertiary care (for example, a specialist stroke unit) from either primary care or secondary care. Tertiary referral centres are usually large hospitals which provide inpatient care (i.e. where you are admitted overnight). |
| Primary healthcare contact | The person you would contact first if you had queries about your condition or treatment. |

### Survey

Question 1

Please indicate whether you are the person living with ataxia or a person completing the survey on their behalf.

*Please check one:*

🞎 Yes, I am the person living with ataxia

🞎 No, I am completing it on behalf of the person with ataxia

Question 2

Please provide your email address if you are happy to be contacted regarding further research and/or would like to receive the results of the survey.

*Please check one:*

- I would like to be emailed regarding further research
- I would like to be emailed the survey results
- I would like to be emailed regarding both further research and the survey results
- I do not wish to provide my email address

Please enter your email address here

Question 3

I confirm that I am (or the person I am completing this survey on behalf of is) aged 16 years or over, live(s) in the UK and wish(es) to proceed with this survey.

*Please check one:*

🞎 Yes 🞎 No

Question 4

Has a healthcare professional ever told you that you have ataxia?

*Please check one:*

- Yes
- No [Please discontinue the survey]
- Unsure [Please discontinue the survey]
- Do not wish to answer [Please discontinue the survey]

Question 5

Which ataxia do you have?

*Please check one:*

- Friedreich’s ataxia
- Inherited cerebellar ataxia
- Cerebellar ataxia (unknown cause)
- Episodic ataxia
- Other [Please specify in the box below]
- Not known
- None [Please discontinue the survey]
- Do not wish to answer

Please enter your response here

Question 6

Have you ever confirmed your final specific ataxia diagnosis e.g. through genetic testing?

*Please check one:*

- Yes, I have a genetic confirmation of my diagnosis e.g. spinocerebellar ataxia type 1 (SCA1) [Please specify in the box below]
- Yes, I have other confirmation of my diagnosis e.g. gluten ataxia [Please specify in the box below]
- No
- Unsure
- Do not wish to answer

Please enter your response here

Question 7

What is your age?

*Please check one:*

🞎16–29 🞎30–59 🞎60–80 🞎80+

Question 8

Which of the following best describes your gender?

*Please check one:*

🞎Female 🞎Male 🞎Not listed 🞎Do not wish to answer

Question 9

Do you have any additional conditions, not related to your ataxia?

*Please check all that apply:*

| - Diabetes mellitus - Liver disease - Malignancy (cancer) - HIV or AIDS - Chronic kidney disease - Congestive heart failure - Myocardial infarction (heart attack) - Chronic obstructive pulmonary disease - Peripheral vascular disease - Cerebrovascular accident or transient ischemic attack (stroke) | - Dementia or Alzheimer’s disease - Hemiplegia (paralysis of one side of the body) - Rheumatic or connective tissue disease - Peptic (stomach) ulcer disease - None - Other [Please specify in the box below] - Not sure - Do not wish to answer |
| --- | --- |

Question 10

Please enter your response here

Do you live alone?

*Please check one:*

- Yes, I live alone
- No, I live with a spouse or with family members
- No, I live with a carer
- Do not wish to answer

Question 11

Which geographical region of the UK do you live in?

*Please check one:*

| - East of England - East Midlands - London - North East & Cumbria - Northern Ireland - North West of England - Scotland | - South East of England - South West of England - Wales - West Midlands - Yorkshire - Other [Please specify in the box below] - Do not wish to answer |
| --- | --- |

Question 12

Please enter your response here

Who do you consider to be your primary contact for ataxia?

*Please check one:*

- General practitioner (GP)
- Neurologist not at a specialist ataxia centre
- Neurologist at a specialist ataxia centre
- Other [Please specify in the box below]
- Unsure
- Do not wish to answer

Please enter your response here

Question 13

Has your ataxia affected your working life?

*Please check one:*

- No, I am still in education
- No, I still work
- No, I have retired
- Yes, I have never worked because of my ataxia
- Yes, I have taken early retirement because of my ataxia
- Yes, I have changed jobs because of my ataxia
- Yes, I have changed to working fewer hours or part-time because of my ataxia
- Other [Please specify in the box below]
- Do not wish to answer

Please enter your response here

Question 14

How long ago did you receive your first general ataxia diagnosis?

*Please check one:*

- Up to 6 months ago
- Between 6 months and 1 year ago
- Between 1 and 2 years ago
- Between 2 and 5 years ago
- More than 5 years ago
- Unsure
- Do not wish to answer

Question 15

Who did you receive your first general ataxia diagnosis from?

*Please check one:*

- General practitioner (GP)
- Neurologist
- Physiotherapist
- Geneticist
- Other [Please specify in the box below]
- Unsure
- Do not wish to answer

Please enter your response here

Question 16

How long did it take from the first time you sought medical advice for your first symptom of ataxia (e.g. visited a GP to discuss your symptom) to the point at which you were referred to a neurologist?

*Please check one:*

- I was referred to a neurologist at the time I first sought medical advice for my first symptom of ataxia
- Up to 6 months
- Between 6 months and 1 year
- Between 1 and 2 years
- Between 2 and 5 years
- More than 5 years
- Unsure
- Do not wish to answer

Question 17

How long did it take from your diagnosis of general ataxia to when you received a specific ataxia diagnosis, if you have received one (e.g. Friedreich’s ataxia, spinocerebellar ataxia type 1 etc.)?

*Please check one:*

- I received the specific ataxia diagnosis at the time I was first diagnosed with ataxia
- Up to 6 months
- Between 6 months and 1 year
- Between 1 and 2 years
- Between 2 and 5 years
- More than 5 years
- I have not received a specific ataxia diagnosis [please proceed to question 19]
- Unsure
- Do not wish to answer

Question 18

How many times did you receive a different non-ataxia diagnosis, e.g. multiple sclerosis, before you received your specific ataxia diagnosis? *Please check one:*

- None
- 1
- 2
- 3
- 4
- 5 or more
- Unsure
- Do not wish to answer

Question 19

When you received your first general ataxia diagnosis, how affected were you by your ataxia?

*Please check one:*

- My ataxia did not affect me
- My ataxia caused occasional problems
- My ataxia caused frequent problems restricting my activities
- My ataxia caused constant problems that restricted me most or all of the time
- Unsure
- Do not wish to answer

Question 20

How many of the following types of appointment did you attend before your first diagnosis of ataxia?

*Please check one answer per line:*

GP appointments

🞎 None 🞎1 🞎 2 to 3 🞎 4 to 5 🞎 More than 5

🞎 Cannot recall 🞎 Do not wish to answer

Hospital outpatient clinic visits with a neurologist (not at a specialist ataxia centre)

🞎 None 🞎1 🞎 2 to 3 🞎 4 to 5 🞎 More than 5

🞎 Cannot recall 🞎 Do not wish to answer

Specialist ataxia centre visits

🞎 None 🞎1 🞎 2 to 3 🞎 4 to 5 🞎 More than 5

🞎 Cannot recall 🞎 Do not wish to answer

Hospital inpatient stays

🞎 None 🞎1 🞎 2 to 3 🞎 4 to 5 🞎 More than 5

🞎 Cannot recall 🞎 Do not wish to answer

Accident and Emergency visits

🞎 None 🞎1 🞎 2 to 3 🞎 4 to 5 🞎 More than 5

🞎 Cannot recall 🞎 Do not wish to answer

Physiotherapist appointments

🞎 None 🞎1 🞎 2 to 3 🞎 4 to 5 🞎 More than 5

🞎 Cannot recall 🞎 Do not wish to answer

Speech and language therapist appointments

🞎 None 🞎1 🞎 2 to 3 🞎 4 to 5 🞎 More than 5

🞎 Cannot recall 🞎 Do not wish to answer

Occupational therapist appointments

🞎 None 🞎1 🞎 2 to 3 🞎 4 to 5 🞎 More than 5

🞎 Cannot recall 🞎 Do not wish to answer

Other consultant specialists (e.g. an ophthalmologist or an ear, nose and throat [ENT] specialist [Please specify the consultant in the box below]

🞎 None 🞎1 🞎 2 to 3 🞎 4 to 5 🞎 More than 5

🞎 Cannot recall 🞎 Do not wish to answer

Please enter your response here

Question 21

In the past six months, how many of the following types of appointment have you attended as a result of your ataxia?

*Please check one answer per line:*

GP appointments

🞎 None 🞎 1 🞎 2 to 3 🞎 4 to 5 🞎 More than 5

🞎 Cannot recall 🞎 Do not wish to answer

Hospital outpatient clinic visits with a neurologist (not at a specialist ataxia centre)

🞎 None 🞎 1 🞎 2 to 3 🞎 4 to 5 🞎 More than 5

🞎 Cannot recall 🞎 Do not wish to answer

Specialist ataxia centre visits

🞎 None 🞎 1 🞎 2 to 3 🞎 4 to 5 🞎 More than 5

🞎 Cannot recall 🞎 Do not wish to answer

Hospital inpatient stays

🞎 None 🞎 1 🞎 2 to 3 🞎 4 to 5 🞎 More than 5

🞎 Cannot recall 🞎 Do not wish to answer

Accident & Emergency visits

🞎 None 🞎 1 🞎 2 to 3 🞎 4 to 5 🞎 More than 5

🞎 Cannot recall 🞎 Do not wish to answer

Physiotherapist appointments

🞎 None 🞎 1 🞎 2 to 3 🞎 4 to 5 🞎 More than 5

🞎 Cannot recall 🞎 Do not wish to answer

Speech and language therapist appointments

🞎 None 🞎 1 🞎 2 to 3 🞎 4 to 5 🞎 More than 5

🞎 Cannot recall 🞎 Do not wish to answer

Occupational therapist appointments

🞎 None 🞎 1 🞎 2 to 3 🞎 4 to 5 🞎 More than 5

🞎 Cannot recall 🞎 Do not wish to answer

Other consultant specialists (e.g. an ophthalmologist or an ear, nose and throat [ENT] specialist) [Please specify the consultant in the box below]

🞎 None 🞎 1 🞎 2 to 3 🞎 4 to 5 🞎 More than 5

🞎 Cannot recall 🞎 Do not wish to answer

Please enter your response here

Question 22

Have you ever needed to stay in hospital overnight because of your ataxia?

*Please check one:*

- Yes
- No
- Unsure
- Do not wish to answer

Question 23

Have you ever been referred to/seen at one of the three specialist ataxia centres (including the Newcastle specialist centre that closed in September 2018)?

*Please check one:*

- Yes
- No [please proceed to question 23i]
- Unsure [please proceed to question 23i]
- Do not wish to answer [please proceed to question 23i]

Question 23a

When were you referred to the specialist ataxia centre?

*Please check one:*

- Up to 6 months ago
- Between 6 months and 1 year ago
- Between 1 and 2 years ago
- Between 2 and 5 years ago
- More than 5 years ago
- Unsure
- Do not wish to answer

Question 23b

Who referred you?

*Please check one:*

- GP
- Other primary care professional
- Hospital neurologist
- Other [Please specify in the box below]
- Unsure
- Do not wish to answer

Please enter your response here

Question 23c

Please rate your experience of the following by checking one box per row:

|  | Very positive | Positive | Slightly positive | Slightly negative | Negative | Very negative | N/A |
| --- | --- | --- | --- | --- | --- | --- | --- |
| Coordinating onward referral to specialists | 🞎 | 🞎 | 🞎 | 🞎 | 🞎 | 🞎 | 🞎 |
| Offers to participate in research | 🞎 | 🞎 | 🞎 | 🞎 | 🞎 | 🞎 | 🞎 |
| Help with benefits | 🞎 | 🞎 | 🞎 | 🞎 | 🞎 | 🞎 | 🞎 |
| Liaising with social workers | 🞎 | 🞎 | 🞎 | 🞎 | 🞎 | 🞎 | 🞎 |
| Help with securing parking badges | 🞎 | 🞎 | 🞎 | 🞎 | 🞎 | 🞎 | 🞎 |

Do you have any further thoughts on the specialist ataxia centres?

Please enter your response here

Question 23d

Do you currently receive care at a specialist ataxia centre?

*Please check one:*

- Yes, I currently receive care at a specialist ataxia centre [please proceed to question 23f]
- No, I was seen at a specialist ataxia centre but no longer receive care there
- Unsure [please proceed to question 24]
- Do not wish to answer [please proceed to question 24]

Question 23e

Why do you no longer receive care at a specialist ataxia centre?

*Please check one:*

- I used to receive care at the Newcastle specialist centre, but the centre is no longer available and I have not been referred to another
- Problems with travelling/transport
- Did not find it useful
- Not referred again
- Equal care locally
- Unable to take time off work to visit the centre
- Other [Please specify in the box below]
- Unsure
- Do not wish to answer

Please enter your response here

Question 23f

How long does/did it take you to travel to visit the specialist ataxia centre?

*Please check one:*

- Less than 1 hour
- 1 to 2 hours
- 3 to 4 hours
- More than 4 hours
- Unsure
- Do not wish to answer

Question 23g

What is the main method of transport that you usually use/used to travel to the specialist ataxia centre?

*Please check one:*

- NHS transport
- Car
- Bus
- Train
- Taxi
- Walk
- Other [Please specify in the box below]
- Unsure
- Do not wish to answer

Please enter your response here

Question 23h

Do you/did you need overnight accommodation when you travel/travelled to the specialist ataxia centre?

*Please check one:*

- Yes
- No
- Unsure
- Do not wish to answer

Question 23i

If you have never been referred to/seen at a specialist ataxia centre, why is this?

*Please check one:*

- Current level of care is sufficient
- I asked to be referred to a specialist ataxia centre but was refused by my doctor
- The specialist ataxia centres are too far away for me to travel to
- Did not wish to be referred
- Other [Please specify in the box below]
- Not applicable
- Unsure
- Do not wish to answer

Please enter your response here

Question 24

How long does/did it take you to travel to receive care that is not at a specialist ataxia centre i.e., at your local hospital?

*Please check one:*

- Less than 1 hour
- 1 to 2 hours
- 3 to 4 hours
- More than 4 hours
- Not applicable
- Unsure
- Do not wish to answer

Question 25

Have you ever been seen by a multidisciplinary team (MDT), including at a non-specialist hospital? Multidisciplinary teams include more than one of the following people: physiotherapists, occupational therapists, orthotics or speech and language therapists.

*Please check one:*

- Yes
- No [please proceed to question 26]
- Unsure [please proceed to question 26]
- Do not wish to answer [please proceed to question 26]

Question 25a

If you have been seen by a multidisciplinary team, including at a non-specialist hospital, how effective did you feel your MDT care was?

| Very Effective | Effective | Slightly Effective | Slightly Ineffective | Ineffective | Very Ineffective |
| --- | --- | --- | --- | --- | --- |
| 🞎 | 🞎 | 🞎 | 🞎 | 🞎 | 🞎 |
| [Please proceed to Question 26] | | | [Please proceed to Question 25b] | | |

Question 25b

What were the reasons for your MDT care being ineffective?

*Please check all that apply*

- There was no referral to a local team in primary care, e.g. a local physiotherapist
- Referral to a local team in primary care, e.g. a local physiotherapist was not effective
- There was no referral to tertiary care (e.g. a specialist urologist)
- Referral to tertiary care (e.g. a specialist urologist) was not effective
- The MDT did not understand my needs
- There was no treatment even after being seen by the MDT
- Other [Please specify in the box below]
- Unsure
- Do not wish to answer

Please enter your response here

Question 26

If you have a genetic diagnosis, have you discussed the implications on insurance or family planning with a health care professional?

*Please check one:*

- Yes
- No
- Not applicable
- Unsure
- Do not wish to answer

Questions 27–38

Please rate your agreement with the following 12 statements on a scale of 1-6:

1. Strongly agree
2. Agree
3. Slightly agree
4. Slightly disagree
5. Disagree
6. Strongly Disagree

|  |  | 1 | 2 | 3 | 4 | 5 | 6 | N/A |
| --- | --- | --- | --- | --- | --- | --- | --- | --- |
| **27.** | Primary care health care professionals (e.g. GP, physiotherapist, occupational therapist) understood how to manage my ataxia. | 🞎 | 🞎 | 🞎 | 🞎 | 🞎 | 🞎 | 🞎 |
| **28.** | Primary health care professionals understood the symptoms of my ataxia. | 🞎 | 🞎 | 🞎 | 🞎 | 🞎 | 🞎 | 🞎 |
| **29.** | Primary health care professionals understood the treatments available for my ataxia. | 🞎 | 🞎 | 🞎 | 🞎 | 🞎 | 🞎 | 🞎 |
| **30.** | Secondary health care professionals (e.g. neurologist, other consultants at my local hospital) understood how to manage my ataxia. | 🞎 | 🞎 | 🞎 | 🞎 | 🞎 | 🞎 | 🞎 |
| **31.** | Secondary health care professionals understood the symptoms of my ataxia. | 🞎 | 🞎 | 🞎 | 🞎 | 🞎 | 🞎 | 🞎 |
| **32.** | Secondary health care professionals understood the treatments available for my ataxia. | 🞎 | 🞎 | 🞎 | 🞎 | 🞎 | 🞎 | 🞎 |
| **33.** | Healthcare workers in Accident & Emergency understood how to manage my ataxia. | 🞎 | 🞎 | 🞎 | 🞎 | 🞎 | 🞎 | 🞎 |
| **34.** | Healthcare workers in Accident & Emergency understood the symptoms of my ataxia. | 🞎 | 🞎 | 🞎 | 🞎 | 🞎 | 🞎 | 🞎 |
| **35.** | Healthcare workers in Accident &Emergency understood how my ataxia might affect the treatment provided to me. | 🞎 | 🞎 | 🞎 | 🞎 | 🞎 | 🞎 | 🞎 |
| **36.** | Specialists at the specialist ataxia centre understood how to manage my ataxia. | 🞎 | 🞎 | 🞎 | 🞎 | 🞎 | 🞎 | 🞎 |
| **37.** | Specialists at the specialist ataxia centre understood the symptoms of my ataxia. | 🞎 | 🞎 | 🞎 | 🞎 | 🞎 | 🞎 | 🞎 |
| **38.** | Specialists at the specialist ataxia centre understood the treatments available for my ataxia. | 🞎 | 🞎 | 🞎 | 🞎 | 🞎 | 🞎 | 🞎 |

Question 39

Are there any other areas of your ataxia that primary health care professionals could have better understood?

Please enter your response here

Question 40

Are there any other areas of your ataxia that primary health care professionals understood well?

Please enter your response here

Question 41

Please rate your agreement with the following statement:

The care I received during the time I spent in A&E could have been better.

| Strongly Agree | Agree | Slightly Agree | Slightly Disagree | Disagree | Strongly Disagree | N/A |
| --- | --- | --- | --- | --- | --- | --- |
| 🞎 | 🞎 | 🞎 | 🞎 | 🞎 | 🞎 | 🞎 |

Question 42

What would your opinion be of patients having a card with key information on your condition for use in A&E?

| A Great Idea | A Good Idea | Not a Bad idea | Not a Good idea | A Bad Idea | A Terrible Idea |
| --- | --- | --- | --- | --- | --- |
| 🞎 | 🞎 | 🞎 | 🞎 | 🞎 | 🞎 |

Question 43

Have you ever been referred for treatment of any of the following symptoms as a result of your ataxia?

*Please tick the box corresponding to your answer in the row opposite each symptom. For example, if you do not wish to answer you would tick box E.*

1. Yes, I have experienced this symptom and have been referred for its treatment
2. No, I have experienced this symptom but have not been referred for treatment
3. No, I have never experienced this symptom
4. Unsure
5. Do not wish to answer

| **Symptom** | A | B | C | D | E |
| --- | --- | --- | --- | --- | --- |
| Pain | 🞎 | 🞎 | 🞎 | 🞎 | 🞎 |
| Heart problems | 🞎 | 🞎 | 🞎 | 🞎 | 🞎 |
| Gastroenterological problems | 🞎 | 🞎 | 🞎 | 🞎 | 🞎 |
| Sexual dysfunction | 🞎 | 🞎 | 🞎 | 🞎 | 🞎 |
| Swallowing | 🞎 | 🞎 | 🞎 | 🞎 | 🞎 |
| Hearing problems | 🞎 | 🞎 | 🞎 | 🞎 | 🞎 |
| Eye symptoms | 🞎 | 🞎 | 🞎 | 🞎 | 🞎 |
| Sleep disturbances | 🞎 | 🞎 | 🞎 | 🞎 | 🞎 |
| Depression | 🞎 | 🞎 | 🞎 | 🞎 | 🞎 |
| Other mental health issues | 🞎 | 🞎 | 🞎 | 🞎 | 🞎 |
| Fatigue | 🞎 | 🞎 | 🞎 | 🞎 | 🞎 |
| Other [Please specify in the box below] | 🞎 | 🞎 | 🞎 | 🞎 | 🞎 |

Please enter your response here

Question 44-49

Have you experienced any of the following symptoms in the past 6 months, and if so how was the symptom treated:

*Please check all that apply*

| **44.** | Stiffness/rigidity of your legs or muscles | - Not applicable. I have not experienced this symptom in the past 6 months - Drug therapy (by mouth or injection) - Physiotherapy - Occupational therapy - Alternative therapies (e.g. homeopathy, acupuncture, reflexology) - Exercise (e.g. visiting the gym, pilates classes) - I have not sought treatment - I have not been offered treatment - Other - Unsure - Do not wish to answer |
| --- | --- | --- |
| **45.** | Cramps | - Not applicable. I have not experienced this symptom in the past 6 months - Drug therapy (by mouth or injection) - Physiotherapy - Occupational therapy - Alternative therapies (e.g. homeopathy, acupuncture, reflexology) - Exercise (e.g. visiting the gym, pilates classes) - I have not sought treatment - I have not been offered treatment - Other - Unsure - Do not wish to answer |
| **46.** | Spasms | - Not applicable. I have not experienced this symptom in the past 6 months - Drug therapy (by mouth or injection) - Physiotherapy - Occupational therapy - Surgery - Alternative therapies (e.g. homeopathy, acupuncture, reflexology) - Exercise (e.g. visiting the gym, pilates classes) - I have not sought treatment - I have not been offered treatment - Other - Unsure - Do not wish to answer |
| **47.** | Bladder problems | - Not applicable. I have not experienced this symptom in the past 6 months - Drug therapy (by mouth or injection) - Catheterisation - Nerve stimulation - Exercise (e.g. visiting the gym, pilates classes) - I have not sought treatment - I have not been offered treatment - Other - Unsure - Do not wish to answer |
| **48.** | Dystonia (uncontrolled and sometimes painful muscle movements causing unusual body positions) | - Not applicable. I have not experienced this symptom in the past 6 months - Drug therapy (by mouth or injection) - Occupational therapy - Physiotherapy - Exercise (e.g. visiting the gym, pilates classes) - I have not sought treatment - I have not been offered treatment - Other - Unsure - Do not wish to answer |
| **49.** | Tremors | - Not applicable. I have not experienced this symptom in the past 6 months - Drug therapy (by mouth or injections) - Occupational therapy - Other - Exercise (e.g. visiting the gym, pilates classes) - I have not sought treatment - I have not been offered treatment - Unsure - Do not wish to answer |

Question 50-55

If you were treated for one or more of these symptoms, who referred you for treatment?

*Please tick one box per row to indicate your answer. For example, if your GP referred you for treatment of cramps, you would tick box B in the second row.*

1. Not applicable. I have not been treated for this symptom in the past 6 months
2. GP
3. Other primary care professional
4. Hospital neurologist
5. Neurologist at a specialist ataxia centre
6. Unsure
7. Do not wish to answer

|  | **Symptom** | A | B | C | D | E | F | G |
| --- | --- | --- | --- | --- | --- | --- | --- | --- |
| 50. | Stiffness/rigidity of your legs or muscles | 🞎 | 🞎 | 🞎 | 🞎 | 🞎 | 🞎 | 🞎 |
| 51. | Cramps | 🞎 | 🞎 | 🞎 | 🞎 | 🞎 | 🞎 | 🞎 |
| 52. | Spasms | 🞎 | 🞎 | 🞎 | 🞎 | 🞎 | 🞎 | 🞎 |
| 53. | Bladder problems |  |  |  |  |  |  |  |
| 54. | Dystonia  (uncontrolled and sometimes painful muscle movements causing unusual body positions) | 🞎 | 🞎 | 🞎 | 🞎 | 🞎 | 🞎 | 🞎 |
| 55. | Tremors | 🞎 | 🞎 | 🞎 | 🞎 | 🞎 | 🞎 | 🞎 |

Questions 56-61-

If you have experienced each of the following symptoms, how well do you feel that the symptom is managed?

|  |  | Very poorly | Poorly | Adequately | Quite well | Very well | Best it could be | N/A |
| --- | --- | --- | --- | --- | --- | --- | --- | --- |
| **56.** | Stiffness/rigidity of your legs or muscles | 🞎 | 🞎 | 🞎 | 🞎 | 🞎 | 🞎 | 🞎 |
| **57.** | Cramps | 🞎 | 🞎 | 🞎 | 🞎 | 🞎 | 🞎 | 🞎 |
| **58.** | Spasms | 🞎 | 🞎 | 🞎 | 🞎 | 🞎 | 🞎 | 🞎 |
| **59.** | Bladder problems | 🞎 | 🞎 | 🞎 | 🞎 | 🞎 | 🞎 | 🞎 |
| **60.** | Dystonia | 🞎 | 🞎 | 🞎 | 🞎 | 🞎 | 🞎 | 🞎 |
| **61.** | Tremors | 🞎 | 🞎 | 🞎 | 🞎 | 🞎 | 🞎 | 🞎 |

Question 62

Overall, including all symptoms mentioned above and any other symptoms that you experience as a result of your ataxia, do you feel like your symptoms are well managed?

| Very poorly | Poorly | Adequately | Quite well | Very well | Best it could be | N/A |
| --- | --- | --- | --- | --- | --- | --- |
| 🞎 | 🞎 | 🞎 | 🞎 | 🞎 | 🞎 | 🞎 |

Question 63

How well do you feel your care reflects your needs?

| Very poorly | Poorly | Adequately | Quite well | Very well | Best it could be | N/A |
| --- | --- | --- | --- | --- | --- | --- |
| 🞎 | 🞎 | 🞎 | 🞎 | 🞎 | 🞎 | 🞎 |

**Question 64**

How do you feel your care could be improved?

*Please check all that apply*

- More information about my condition
- More information about available treatments
- More help to make me feel in control of my condition (i.e. to cope better)
- Knowing my specific diagnosis earlier
- Better management of my symptoms
- Better practical advice on living with the condition
- Better access to therapies (e.g. physiotherapy, speech and language therapy, occupational therapy)
- More information on help adapting my home
- Help communicating with my employer
- More information on the genetics of my condition/whether my children or grandchildren would be at risk of inheriting ataxia
- Continuing the same level of care in my home if I cannot attend a SAC any longer
- I am satisfied with my care, it does not need improvement
- Other [Please specify in the box below]
- Unsure
- Do not wish to answer

Please enter your response here
